# The Role of Gut Microbiota in Overcoming Resistance to Checkpoint Inhibitors in Cancer Patients: Mechanisms and Challenges

**DOI:** 10.3390/ijms22158036

**Published:** 2021-07-27

**Authors:** Youssef Bouferraa, Andrea Chedid, Ghid Amhaz, Ahmed El Lakkiss, Deborah Mukherji, Sally Temraz, Ali Shamseddine

**Affiliations:** Department of Internal Medicine, Division of Hematology/Oncology, American University of Beirut Medical Center, Riad El Solh, Beirut 1107 2020, Lebanon; Youssef.bf95@gmail.com (Y.B.); ac81@aub.edu.lb (A.C.); ga116@aub.edu.lb (G.A.); ae147@aub.edu.lb (A.E.L.); dm25@aub.edu.lb (D.M.); st29@aub.edu.lb (S.T.)

**Keywords:** microbiota, immune checkpoint inhibitors, gut microbiota, immune system, dysbiosis

## Abstract

The introduction of immune checkpoint inhibitors has constituted a major revolution in the treatment of patients with cancer. In contrast with the traditional cytotoxic therapies that directly kill tumor cells, this treatment modality enhances the ability of the host’s immune system to recognize and target cancerous cells. While immune checkpoint inhibitors have been effective across multiple cancer types, overcoming resistance remains a key area of ongoing research. The gut microbiota and its role in cancer immunosurveillance have recently become a major field of study. Gut microbiota has been shown to have direct and systemic effects on cancer pathogenesis and hosts anti-tumor immune response. Many studies have also shown that the host microbiota profile plays an essential role in the response to immunotherapy, especially immune checkpoint inhibitors. As such, modulating this microbial environment has offered a potential path to overcome the resistance to immune checkpoint inhibitors. In this review, we will talk about the role of microbiota in cancer pathogenesis and immune-system activity. We will also discuss preclinical and clinical studies that have increased our understanding about the roles and the mechanisms through which microbiota influences the response to treatment with immune checkpoint inhibitors.

## 1. Introduction

**Figure 1:**
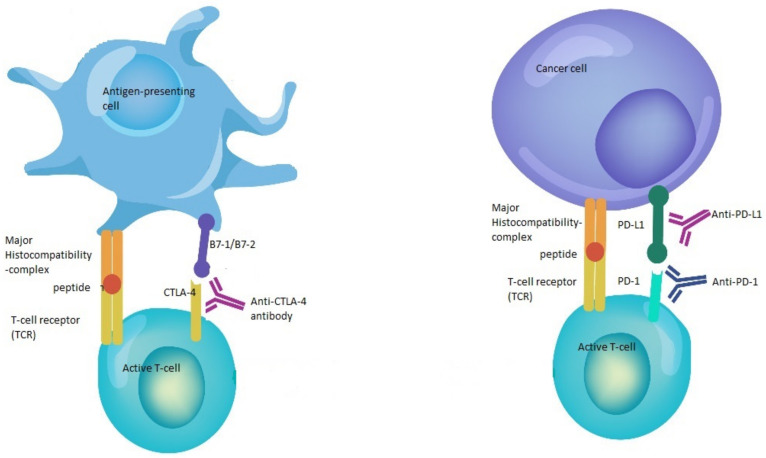
Mechanisms of action of immune checkpoint inhibitors. The binding of B7-1/B7-2 to CTLA-4 keeps the T cells in the inactive state so that they are not able to kill tumor cells in the body. Blocking the binding of B7-1/B7-2 to CTLA-4 with anti-CTLA-4 antibody allows the T cells to be active and to kill tumor cells. In the same way, the binding of PD-L1 to PD-1 prevents the T cells from killing cancer cells. The interruption of this binding using anti-PD-1/PD-L1 enhances the ability of T cells to kill tumor cells.

Immune checkpoint inhibitors (CPI) constitute one of the major advances in immunotherapy and cancer treatment [1]. This family of drugs is directed against immune checkpoints, such as programmed cell death 1 (PD-1), PD ligand 1 (PD-L1) and cytotoxic T-cell lymphocyte-associated protein (CTLA-4) [2] (Figure 1). These checkpoints are part of a delicate system of stimulatory and inhibitory proteins that tightly control the T- cell immune response through the regulation of cytotoxic T-lymphocyte activation, maintenance of self-tolerance, prevention of autoimmunity, and adjustment of the duration and amplitude of the immune response in order to reduce tissue damage during a period of inflammation [3,4,5,6]. Nonetheless, tumor cells have been shown to exploit these inhibitory checkpoints in order to evade the immune system [7].

Several CPIs have been approved for use in clinical practice, demonstrating prolonged overall survival (OS) and improved safety profile in cancer patients when compared with other treatments in various types of tumors, such as metastatic melanoma, non-small cell lung cancer (NSCLC), renal cell carcinoma (RCC) and urothelial carcinoma [3,4,8,9,10]. However, despite these encouraging findings, the success rate of CPIs remains limited, with only a fraction of patients with advanced disease having long-term benefit [7,11]. As such, and given the significant financial cost associated with novel CPIs, it is of great interest to identify patients who are most likely to benefit from these therapies by establishing biomarkers that can predict a positive response to treatment and a durable clinical benefit.

In this regard, a high tumor mutational burden (TMB) has been identified as an important biomarker associated with an improved response rate and survival benefit from PD1/PD-L1 blockers [12]. In addition, tumors with defective DNA repair mechanisms have a higher probability of benefiting from CPIs [13]. Moreover, tumor microenvironment (TME) has been shown to play a role in the response to treatment with CPIs. By definition, TME is a complex network of cells that surrounds tumor cells, modulates the innate and adaptive immune response, and affects tumor growth in response to treatment [14,15]. Several factors were shown to directly and indirectly modulate the TME. For example, alterations in energy metabolism appear to influence immune cells in the TME. This is due to the fact that cancer cells increase the consumption and consequently, limit the nutrient abundance for immune-infiltrating cells, impairing effector T-cell activation and stimulating regulatory immune cells instead, resulting in CPI resistance [16,17]. In addition, obesity, gender, diet and smoking habits were also shown to influence the TME and consequently, impact the response of patients with cancer to CPIs [18,19,20,21,22,23,24].

More recently, the gut microbiota has been surfacing as a potential predictor of response to CPIs [25]. Studies have demonstrated that gut microbiota plays a major role in immunosurveillance and positively impacts the efficacy of CPIs [26,27,28,29,30]. In this review, we will talk about the role of microbiota in cancer pathogenesis and immune-system activity. We will also discuss the preclinical and clinical studies that increased our understanding about the role of microbiota in influencing the response to treatment with immune checkpoint inhibitors, focusing on the mechanisms through which this microbiota could help in overcoming the witnessed resistance to immune checkpoint inhibitors.

## 2. Role of Gut Microbiota in Cancer Development and Immunosurveillance

**Figure 2:**
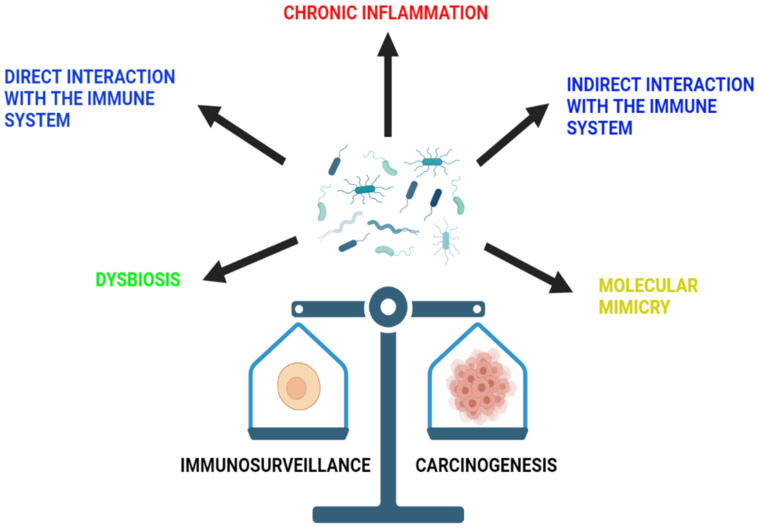
The role of microbiota in immunosurveillance and carcinogenesis. The microbiota of the gut is able to influence immunosurveillance and carcinogenesis through a variety of mechanisms. These include chronic inflammation, dysbiosis and direct and indirect interaction with the immune system, as well as molecular mimicry.

The microbiota of the human body includes a diversity of bacteria, viruses, fungi, and protozoa that usually exist on the different epidermal and mucosal surfaces of the body. These microbiota are essential elements of human health, as they play an important role in the control of different systemic functions [31]. They mostly exert their effect by inducing the synthesis of short chain fatty acids (SCFA) from dietary fibers, as well as different vitamins, including vitamin B and vitamin K [32]. In addition, they also work on the breakdown of multiple complexes, such as sterols and xenobiotics, consequently boosting the immune system [32]. It was described that microbiota plays an important role in cancer, liver diseases, obesity, and neuropsychiatric disorders [31]. In fact, many infectious organisms were found to be causative elements in the development of different cancers. For example, *Helicobacter pylori* was associated with gastric cancer development [33]. In addition, the DNA of *Fusobacterium nucleatum* was detected in the tumor cells of colorectal adenomas and cancers that work through the Wnt signaling cascade [34]. This correlation is explained by a pathogen-induced alteration in the host environment that would facilitate the path of the host cells to becoming neoplastic [35]. However, the association between the microbiota and cancer development varies among organs [35]. Below is a list of mechanisms through which the microbiota influences immunosurveillance and carcinogenesis (Figure 2).

### 2.1. Dysbiosis

Dysbiosis is characterized by the disruption of gut microbial homeostasis, leading to an imbalance in microbiota diversity, composition, function, distribution, and activity [36]. This dysbiosis was shown to be associated with different mutations in the host’s genes, consequently influencing the immune system by altering innate immunity [37]. In addition, dysbiosis of the gut microbiota was clinically linked to the development of many types of cancer, including colorectal cancer (CRC) [38]. 

It is interesting to know that dysbiosis is not only induced by pathogenic organisms [39]. Aging, antibiotics, xenobiotics, smoking, hormones, and dietary elements can also lead to this disruption and consequently, constitute risk factors for CRC [39]. It is also noticed that factors promoting inflammation through different genetic defects affecting epithelial, myeloid, or lymphoid components of the intestinal immune system are carcinogenic through the induction of dysbiosis [39]. Thus, nowadays we know that tumor driver mutations can be regulated by the microbiota [40]. 

### 2.2. Chronic Inflammation

Another mechanism through which microbiota can induce carcinogenesis is the induction of chronic inflammation. Inflammation plays an important part in the pathogenesis of cancer [40]. A chronic inflammatory state exists in multiple conditions, including inflammatory bowel disease, pancreatitis, and chronic atrophic gastritis, and was linked to the development of cancer through several mechanisms [41]. In the same way, inflammation caused by microbes also favors carcinogenesis [40]. Examples include *H. pylori* as a causative agent of gastric cancer and *Schistosoma haematobium* infection as a risk factor for bladder cancer [40].

### 2.3. Direct Interaction with the Immune System

More evidence is coming up to emphasize the ability of the microbiota to anticipate malignancy, manipulate the reaction to immunotherapy and even correlate with survival outcomes in certain cancers [40]. In the 19th century, it was shown that chicken sarcoma virus was able to cause sarcoma in healthy chickens [42]. This was the first proven correlation between the host microbiota, immune system, and cancer [40]. Germ-free animals were the first to be used to investigate this connection [40]. These models lack mucosal immunity because of deficits in Peyer’s patches, mesenteric lymph nodes, lymphoid follicles in the lamina propria, pattern recognition receptors (PRRs), such as Toll-like receptors (TLRs), and the excessive activation of anti-inflammatory T helper (Th) type 2 cytokines [43]. With microbiota implantation in these models, such deficits were replaced, consequently helping in the development of an innate and adaptive immune system [40]. In fact, IL-4 and TGF-ß promote the differentiation of a subset of CD4-T cells called T-helper (Th) 9 cells and induce the secretion of IL-9, which is a key element in antitumor immunity [40]. It was noticed that the GF models lacked Th9, which wereinduced and restored after the implementation of microbiota [44]. One of the major microbiota that was noticed to increase this CD4+ maturation in GF mice was *Bacteroides fragilis* [40]. Similarly multiple microorganisms were noticed to induce IFNg+ CD8 T-cell3 [40]. Moreover, the specificity and diversity of B cell collections in GF mice was also shown to be determined by the site of microbial exposure [40]. IgA production was mainly induced by transient mucosal exposure, whereas IgM and IgG depended on systemic exposure so they can be able to face invasive infections [45]. This shows that each immune system of each host is unique and influenced by their microbiota exposure [40]

This correlation between microbiota, immune system and carcinogenesis was further investigated with time. The microbiota was proven to induce carcinogenesis through the direct interaction with immune cells. For example, *Fusobacterium* promotes the growth of colorectal cancer through variable direct mechanisms, including the suppression of the immune system. This is possible through the inhibition of T-cell activity and NK cell cytotoxicity and the induction of the activity of myeloid suppressor cells and tumor-associated macrophages (TAMs), in addition to the inhibition of the tumor-infiltrating lymphocytes in MSI-high types of colorectal cancer [46]. Here rises the relationship between the microbiota, the immune system and cancer progression through immune cells interactions [47]. 

The first cells to fight microbes are the dendritic cells that lay in lamina propria [47]. Through their pattern recognition receptors, they recognize microbes and present them to the adaptive immune system through antigen-presenting cells [47]. Gut microbiota tend to produce butyrate, which in turn can inhibit the dendritic cells’ antigen presentation, consequently decreasing the CD8+ T-cell response [47]. 

Natural killer T-cells, which also carry a cytotoxic activity as part of the innate immune system, are also influenced by the gut bacteria. In fact, by inducing the metabolism of primary bile acids into secondary bile acids, gut microbiota is able to inhibit the immune-system surveillance of liver tumors by decreasing the CXCR6+ NKT cells through the pattern of C-X-C motif chemokine ligand -16(CXCL)-16 [48].

In addition, tumor growth and micrometastasis can also be induced by TAMs [49]. M2 polarized macrophages aim to suppress the TME through the secretion of chemokines and cytokines [49]. Through dysbiosis, discussed above, and through Cathepsin K (CTSK)-mediated TLR4 signaling, microbiota can induce the proliferation of this M2 phenotype of macrophages and consequently, induce tumor growth and metastasis [50]. Following the same pathway, the intra-tumoral bacteria also provoke the proliferation of the M2 phenotype through the TLR2 and TLR5 in pancreatic ductal adenocarcinoma (PDAC) [50]. Through the TLR5 pattern and mainly through the increase in systemic IL-6 secretion, bacteria were able to provoke malignant progression, mainly at extra mucosal sites [51]. This is how *Fusobacterium* plays a role in colorectal cancer progression. In fact, this species can induce the suppression of the T-cell receptors, as previously discussed [52]. 

Furthermore, follicular T helper cells (TFH) mainly reside in the mucosal lymphoid tissue and function through IL-1R and IL-12 to decrease tumor size [52]. The microbiota in the ileum can have variable effects in this regard, with some acting as stimulators for TFH cells and others as tolerogenic yet not as tumorigenic [52]. In addition, gut microbiota is important for the development of IL-9-secreting cells, which play an important role in decreasing tumor growth [52]. 

Moreover, CD4+ and CD8+T cells, which tend to secrete interferon (INF)-γ, can be specific for microbial epitopes and consequently, may lead to an increase in INF- γ and a decrease in IL-10 and IL-17 upon microbiota exposure modulating the immune response [46]. γδ T cells are a subset of T cells that do not depend on MHC molecules for antigen presentation, yet they express TCRs and consequently, they are able to induce an immune response [53]. The microbiota was shown to induce the production of Vγ6+ Vγ1+ γδ T cells through the Myd88 signaling pathway, provoked by IL-17 production, and hence activate the immune system in the TME [53]. 

### 2.4. Indirect Interaction with the Host Cells and Immune System

**Figure 3:**
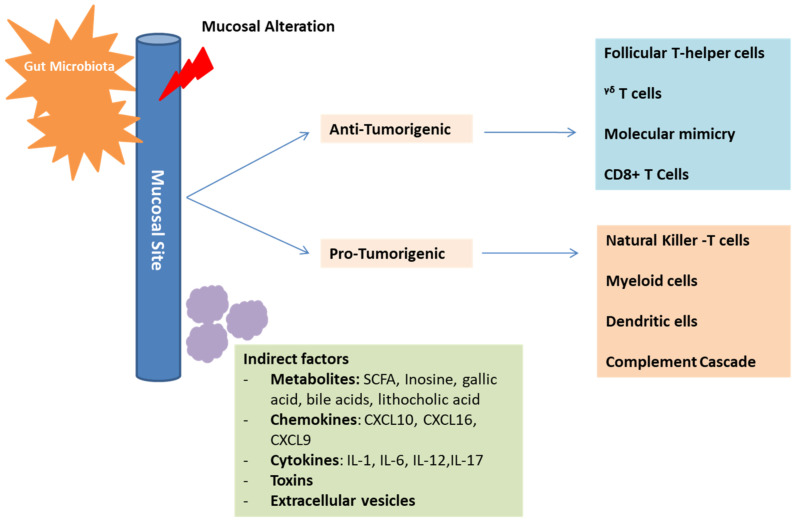
Mechanisms of gut microbiota in cancer development and immunosurveillance. The gut microbiota plays an essential role in influencing the immune-system composition and function. This is possible through direct, as well as indirect interactions with the immune-system components. These interactions can have positive anti-tumorigenic effects on some cells and negative immunosuppressive pro-tumorigenic effects on other cells, depending on the established microbiota profile. Mediators of indirect interactions include metabolites, chemokines, cytokines, toxins, and extracellular vesicles.

In addition to the direct interaction mentioned above, an indirect interaction also allows the microbiota to influence the immune response and cancer progression (Figure 3). 

This is possible through different mediators [29]. For example, *Escherichia coli* can produce a product called colibactin, which in turn can induce the alkylation of the DNA on the adenine residue, inducing double-strand breaks [54]. This will provoke a carcinogenic effect similar to that found in colorectal cancer [54]. In addition, there is an emerging evidence indicating that, through the microbial metabolite gallic acid, the microbiota can harbor oncogenic properties of genetic mutations similar to *Tp53* [55]. Other metabolites that harbor oncogenic properties as well include lithocholic acid, SCFA, cadaverine and de-conjugated estrogens, which mainly contribute to the pathogenesis of breast cancer [56]. As for SCFA, they tend to inhibit the histone deacetylase (HDAC), which plays a role in regulating the innate immune system, controlling myeloid cell differentiation, and modulating inflammatory responses controlled through TLR- and INF- gene expressions [56]. In fact, it was shown that the stools of patients responding to immunotherapy have a good amount of SCFA in contrast to those showing no response [57]. This can emphasize the fact that the inhibition of HDAC can play a role in the upregulation of the expression of the PDL-1 and PDL-2 and thus, work in line with anti-PDL1 therapy [56]. In addition, the butyrate limits CD80/CD86 regulation and thus, increases the effectiveness of the anti-CTLA-4 treatment [58]. As a secondary bile acid, deoxycholic acid is also incorporated in the pathogenesis of hepatocellular carcinoma [40]. Moreover, lipoteichoic acid, coming mainly from gram-positive bacteria, activates the production of TLR-2 dependent prostaglandin E2 that aims at suppressing the immune system in the TME [59]. Inosine, a purine metabolite mainly secreted by *Bifidobacterium pseudolongum, Lactobacillus johnsonii* and *Olsenella* species was also found to play an important role in immune checkpoint blockade [60]. Inosine and its metabolite hypoxanthine translocate into the systemic circulation and provoke the differentiation of the Th1 cells through the inosine-A2AR-cAMP-PKA pathway [60]. It also functions as a carbon source for T-cells supporting their differentiation and proliferation and consequently, enhances the response to CPIs [40,60]. Mannose binding lectin (MBL), a metabolite of the *Malassezia* species, leads to the activation of complement C3 and provokes an oncogenic effect [61]. Finally, another mode of indirect interaction between the host and the gut microbiota takes place through extracellular vesicles. These vesicles mainly send signaling molecules, metabolites or other antigenic proteins that would induce an anti-inflammatory response or a pathological process [62]. 

### 2.5. Molecular Mimicry

Molecular mimicry (or direct antigenicity) is also an important mechanism through which the microbiota influences immune-system surveillance and potentially contributes to carcinogenesis [29]. It was shown that multiple microbial epitopes are similar either to host antigens or to tumoral antigens, impacting the development of autoimmune diseases, as well as the development and treatment of different malignancies [29,40]. This cross-reactivity affects the efficacy of anticancer immunotherapy, as the CD4+ and CD8+ T cells that are specific for the microbial epitopes are important for the effectiveness of anti-PD1 and anti-CTLA4 treatments [29]. 

## 3. Microbiota and Immunotherapy in Preclinical Studies

The importance of the microbiota as a distinctive influencer of cancer immunotherapy, most notably anti-PD-1/PD-L1 and anti-CTLA-4, has been the focus of numerous published and ongoing studies. However, the complete interpretation of the underlying mechanisms behind these interfaces has not yet been attained. Preclinical mouse models have formed an imperative tool for investigating the prospect of gut microbiota modulating response to CPIs. This section reviews the notable studies that have explored the correlation between the microbiota and CPIs, as well as the underlying mechanisms of action in murine models, with highlights on the potential therapeutic strategies in progress.

### 3.1. Anti-PD-1/PD-L1

A pioneering study led by Sivan et al. in 2015 found that genetically similar mice C57BL/6 mice, housed at different vendors, Jackson Laboratory (JAX) and Taconic Farms (TAC), had dissimilar baseline microbiota that showed varying T-cell-mediated anti-tumor responses to melanoma [13]. This led researchers to further investigate the role of commensal microbiota in this regard. Cohousing of the two mouse populations JAX and TAC abolished the difference in tumor growth [13]. Subsequently, the anti-tumor effects of microbiota were confirmed after the administration of fecal material by oral gavage, alone or in combination with PD-1 antibodies, from responder (JAX) to non-responder (TAC) tumor-bearing mice [13]. The transfer of fecal material resulted in significantly slower tumor growth, spontaneous immune-mediated tumor control, an increase in tumor-specific T cell responses and a subsequent infiltration of the tumor by antigen-specific T cells, to a similar degree as the PD-L1 targeted antibody treatment [13]. Furthermore, *Bifidobacterium* was identified as a bacterium of interest. *Bifidobacterium*-treated mice by oral gavage exhibited significantly enhanced tumor control compared to their non-*Bifidobacterium*-treated counterparts [13]. This was associated with the upregulation of gene transcripts involving DC maturation, CD8+ T-cell activation and co-stimulation, and antigen processing-mediated immune cell recruitment to the TME [13]. In addition, increases in major histocompatibility complex Class II^hi^ DC in responders’ tumors and INF-γ-producing tumor-antigen-specific T-cells were noted [13]. Of note, the systemic immune responses ensued are independent of *Bifidocaterium* translocation, since this bacterium was not detected in either the tumor, the mesenteric lymph nodes, or the spleen [13].

In another study published in 2018, researchers investigated the association between a “favorable” gut microbiota and response to CPIs in germ-free recipient mice bearing melanoma [27]. Fecal material samples were collected from responding (R-FMT) and non-responding (NR-FMT) human subjects to anti-PD-1 immunotherapy [27]. Consequently, mice transplanted with R-FMT displayed an improved anti-tumor response with PD-L1 antibodies therapy and a significant decrease in tumor size (*P* = 0.04), in contrast to mice transplanted with stool from non-responders. Further correlative studies done to better apprehend the fundamental mechanism through which gut microbiota may impact systemic and anti-tumor immunity demonstrated that mice receiving R-FMT had higher levels of CD8+ T cells in their tumors as well as an increase in the number of CD45^+^ immune and CD8+ T cells in their guts [27]. Moreover, the upregulation of PD-L1 was denoted in the tumor microenvironment (TME) of responsive mice as compared to their non-responsive counterparts, which is suggestive of the development of a “hot” tumor microenvironment [27]. Furthermore, a substantial enhancement of innate effector cells (expressing CD45^+^CD11b^+^Ly6G^+^) and reduced the concentration of suppressive myeloid cells (expressing CD11b^+^CD11c^+^) were detected on phenotypic studies of tumor immune infiltrates in mice with favorable gut microbiota [27]. Finally, mice transplanted with NR-FMT stool disclosed a rise in RORγT^+^ T helper 17 tumor-infiltrating cells and higher densities of regulatory CD4^+^FoxP3^+^ T cells and CD4^+^IL-17^+^ T cells in the spleen, indicating a compromised immune response by the host [27].

Tanoue et al. isolated IFNγ+ CD8 T-cell-inducing human bacteria strains [63]. The identified bacteria were 11 strains: *Ruthenibacterium lactatiformans*, *Eubacterium limosum*, *Fusobacterium ulcerans*, *Phascolarctobacterium succinatutens*, *Bacteroides uniformis*, *Bacteroides dorei*, *Paraprevotella xylaniphila*, *Parabacteroides distasonis*, *Parabacteroides johnsonii*, *Parabacteroides gordonii* and *Alistipes senegalensis* [63]. MC38 adenocarcinoma colon tumors in mice responded to targeted anti-PD-1 or anti-CTLA-4 antibodies; however, this response was impaired in the gnotobiotic mice group or with the administration of antibiotics prior to immunotherapy [63]. Oral gavage of the human gut bacterium (11-mix) established a recovery of the response to immune checkpoint inhibitor therapy, with an induction of infiltrating INFγ+ CD8 T-cells in tumors [63]. These 11 strains comprised 7 *Bacteroidales* and 4 non-*Bacteroidales* species in a phylogenetic comparison. Germ-free mice were then inoculated: the 4 non-*Bacteroidales*-mix (4-mix) displayed a significant augmented capacity in inducing INFγ+ CD8 T-cells compared to the other microbial mix [63]. However, an insufficient inductive effect was seen when 4-mix was used alone, compared to the 11-mix. These results indicate that the 4 non-*Bacteroidales* species function as effector components, and the 7 *Bacteroidales* have a supplementary role, and thus the 11 strains operate in consortium [63].

Similarly, Routy et al. transplanted mice with established sarcomas (MCA-205) with FMT by oral gavage from different non-small cell lung cancer (NSCLC) patients, responders R and non-responders NR [30]. The “avatar” mice with FMT from R patients revealed a delay in tumor growth upon treatment with anti-PD-1 as compared to a resistance to treatment seen in the other mouse model group [30]. Additionally, the TME was enriched with CXCR3^+^CD4^+^ T cells, and the upregulation of PD-L1 expression in the T cells within the spleen was observed after PD-1 blockade [30]. Probiotics supplementation with *A. muciniphila* alone or in combination with *E. hirae* was also performed in antibiotics-treated RET melanoma-bearing mice reared in specific-pathogen-free (SPF) conditions. Mono-supplementation with *A. muciniphila* or dual supplementation of *A. muciniphila* plus *E. hirae* reversed the PD-1 blockade resistance that was previously instigated by the antibiotics-induced dysbiosis [30]. On the cellular level, the introduced microbiota induced the formation of intra-tumoral granulomas, the secretion of IL-2 by dendritic cells (DC) and the accumulation of central memory (TCM) CD4^+^ T cells in tumor beds, as well as mesenteric and tumor-draining lymph nodes after 48 h of the first injection of anti-PD-1 therapy [30]. In a similar translational hybrid model, germ-free (GF) recipient mice were gavaged with fecal material from responder and non-responder human patients and inoculated with B16.SIY melanoma cells after two weeks [64]. Two-thirds of the mice group colonized with responders FMT and one-third of non-responders FMT recipient mice group revealed a slower rate of tumor growth when administrated in combination with anti-PD-L1 antibody therapy [64]. Mechanistic studies presented a significant larger number of SIY-specific CD8^+^ T cells but not of FoxP3^+^CD4^+^ regulatory T cells in the tumor microenvironment of mice with “beneficial” commensals, which is consistent with the upturn priming of the potential mediator, tumor antigen-specific CD8^+^ T cells. Moreover, splenic IFNγ^+^CD8^+^ T-cells and Batf3^+^ DCs were increased [64].

Lee et al. identified that *B. bifidum* species was significantly abundant in responder NSCLC patients, whereas *A. Muciniphila* and *Blautia obeum* were in abundance in non-responders [65]. Researchers employed murine models of syngeneic MC38 colon and Lewis lung carcinoma (LLC1) tumors to determine the potential modulation of the response to cancer therapeutics by *B. bifidum*. Subsequently, four commercial *B. bifidum* strains were used. Only specific *B. bifidum* strains (*B. bif*_K57) conveyed a synergistic association with anti-PD-1 to decrease tumor growth [65]. A reduced efficacy of targeted PD-1 antibody therapy was seen in antibiotics-treated mice. However, mice reconstituted with *B. bif*_K57 plus anti-PD-1 after antibiotics treatment displayed a significant decline in tumor growth compared to antibiotics-treated mice that received anti-PD-1 alone [65]. The inconsistencies in therapeutic effectiveness were complemented with systemic and tumor immune modulation. Mice treated with synergistic *B. bifidum strains (B. bif*_K57) and anti-PD-1 manifested an increase in cytokine-producing IL-2^+^CD4^+^ and IFN-γ^+^CD8^+^ tumor-infiltrating T cells, anti-tumor lymphocytes CD4^+^ T, CD8^+^ T, NK cells, CD8^+^ T/Treg cells and effector CD8^+^ T/Treg cell ratios in the tumor and the spleen [65]. Additionally, RNA sequencing was performed on collected intestinal tissues from mice treated with synergistic *B. bifidum* strains and anti-PD-1 therapy. Analysis showed a significant increase in genes correlated with lymphocyte activation, positive regulation of interferon-gamma production and peptidoglycan synthesis [65]. Interestingly, the injection of INF-γ receptor (INF-γ R) antibody into syngeneic mice abolished the tumoricidal effects of both *B. Bif*_K57 plus anti-PD-1 and anti-PD-1 alone [65]. Metabolomics profiling showed an increase in serum L-tryptophan levels in mice treated with *B. bif*_K57 and anti-PD-1 compared to mice treated with *B. bif*_B06 and anti-PD-1. Thus, INF-γ production and signaling are the means of anti-tumor immunity for *B. bifidum* strains, associated with increased L-tryptophan serum levels and peptidoglycan synthesis [65]. Moreover, serum lipid levels were lower in mice treated with *B. bifidum* relative to mice treated with anti-PD-1 alone [65]. This result supports several studies that have described the lipid-lowering characteristics of *Bifidobacterium* spp. and the potential consequences of lipids on immune cells [66,67]. Therefore, *B. bifidum* accomplishes its anti-tumor effects via various mechanisms, and lipid lowering can be one of them [65].

Xu et al. investigated the cause–effect link between gastrointestinal microbiota and PD-1 antibody treatment in MSS-type colorectal cancer murine models (CT26 CRC), as well as the comparative efficacy of different antibiotic groups on immunotherapy [68]. Mice were divided into four groups, each group receiving a specific antibiotic cocktail: Group 1 received a mix of ampicillin, streptomycin and colistin (Asc group); Group 2 received vancomycin alone (Vanc group); Group 3 received colistin alone (Coli group), and Group 4 was the control group and thus, was treated with sterile drinking water. Results showed that the poor anti-tumor response of PD-1 antibodies therapy was seen in the Coli group, as compared to a “well-response” in the Control group, medium response in the Vanc group and no response in the broad-spectrum-treated mice, the Asc group [68]. Moreover, 16S rRNA gene sequencing and metagenomic whole-genome shotgun (WGS) sequencing revealed an abundance of Bacteroidales_S24–7 in the Control group, *A. muciniphila* in the Vanc group and *Bacteroides* in the Coli group [68]. The finding of *A. muciniphila* mirrors the outcome of the study conducted by Routy et al. [30,68]. Commensals altered the expression of immune-related cytokines INF-γ and IL-2 in all groups except the control group. Thus, microbiota were able to prime anti-PD-1 therapy via INF-γ immunoregulatory and tumoricidal effects [68]. Interestingly, researchers utilized the KEGG pathway database to determine the functional diversity of gut microbiota. They discovered that metabolic and synthetic metabolic pathways predominated in the Vanc group, including glycosphingolipid bio-synthesis-globo, glycerolipid and sphingolipid metabolism and the PPAR signaling pathway. Hence, “favorable” gut microbiota, such as a high abundance of *A. Muciniphila,* enhances anabolic functions and thus, contributes to anti-tumor and systemic responses [68]. Conversely, the enrichment of the Type I diabetes mellitus and immune-related pathways, such as IL-17 signaling pathway and Th17 cell differentiation, was seen in the Coli group, which may account for the deprived efficacy of immunotherapy [68].

In a hybrid study design, Dees et al. generated humanized microbiota murine model [69]. Fecal samples were derived from healthy human donors and then transplanted via oral gavage into GL261 brain-tumor-bearing gnotobiotic mice, sharing the same genetic strain [69]. HuM1–HuM5 were the humanized-microbiota bearing mice, and MuM were the controls with murine microbiota only. HuM2 and HuM3 mice exhibited a strong positive response to PD-1 antibody therapy, manifested by a slower rate of tumor growth and prolonged survival [69]. However, HuM1, HuM4 and HuM5 displayed resistance to immunotherapy. On further analysis of cytokine expression and T-cell population percentages, responder mice HuM2 demonstrated a significant enrichment in INF-γ production, increase in cytotoxic CD8^+^ and CD4^+^ T cells and the CD8^+^/Treg ratio as compared to resistant HuM1 mice [69]. Taxonomic distribution analysis of microbial communities showed close clustering of the HuM2 and HuM3 microbes, indicating similarities in composition and the potential underlying mechanism to enhance therapeutic effectiveness [69]. *Bacteroides cellulosilyticus* were found in abundance in these two groups, and *B. caccae* specifically in HuM2. Treatment with antibiotics prevented PD-1 antibody therapy efficacy in HuM2, hence the vital role of HuM2 microbes in modulating immune response [69].

Nowadays, alternative and complementary supplements are gaining more attention. Traditional Chinese medicine (TCM) was investigated in multiple studies. A classical TCM formula, Gegen Qinlian decoction (GQD), was shown to augment the anti-tumor effect of anti-PD-1 inhibition therapy in colorectal murine model [70]. Gegen Qinlian decoction promoted the regulation of the glycerophospholipid metabolic pathway, thus modifying intestinal microbiota and increasing the expression of IL-2 and INF-γ in tumors, which ensured an enhancement of anti-PD-1 therapy [70]. Moreover, a combination of GQD- and PD-1-targeted antibodies led to the relative growth and abundance of *Lactobacillus* and *Sutterella,* with downregulation of *Bacteroides*, which could impede tumor growth in B16 tumor-bearing mice [71]. The bilberry anthrocyanins microbiota-stimulating effects were explored by Liu et al. [72]. They unraveled that anthrocyanins/anti-PD-L1 combo achieved the highest efficiency in treatment against colon cancer in murine models [72]. Anthrocyanins stimulated the overpresentation of *Lachnospiraceae* and *Ruminococcaceae*, which are butyrate-producing species [72]. Increased SCFAs production, especially butyrate, exerted a vital role in promoting a better control of tumor growth [72]. Anthrocyanins enriched the immune cells’ infiltration of the tumor with heightened CD8^+^ T cells proportion [72].

### 3.2. Anti-CTLA-4

In a study by Vetizou et al., MCA205 sarcomas-bearing mice treated with CTLA-4 antibody therapy manifested varying anti-tumor responses relative to the housing conditions [73]. Germ-free and antibiotics-treated mice experienced a compromise in their immunologic response against tumors, with a significant decrease in tumor-infiltrating lymphocytes and effector CD4^+^ T-cells compared to the control group [73]. Recovery of an efficient tumoricidal response to anti-CTLA-4 therapy was established by oral feeding with *Bacteroides* spp. or *Burkholderia* spp. [73]. This recovery was correlated with the maturation of intratumoral dendritic cells and TH1 immune responses in tumor-draining lymph nodes [73].

In colon cancer syngeneic mouse models, researchers revealed that the co-administration of *L. acidophilus* cell lysates with anti-CTLA-4 therapy significantly protected against tumor development [74]. The improved effectiveness to immunotherapy was correlated with the immune-modulatory effects of *L. acidophilus* probiotics. These include an increase in CD8+ T-cells and effector memory T cells (CD44^+^ CD8^+^ CD62L^+^) and a decrease in Treg cells (CD4^+^ CD25^+^ Foxp3^+^) and M2 macrophages in the tumor microenvironment [74]. Moreover, 16S rRNA gene sequencing of gut microbiota showed that a lysates/CTLA-4 antibodies combination significantly hindered the overpresentation of proteobacteria and partially compensated for CRC-induced dysbiosis [74].

Table 1 summarizes the main preclinical studies involving microbiota and CPIs. The inconsistencies across studies highlight multiple concerns. The immune-modulating effects of gut microbiota are different according to the study design, especially if a translational hybrid model is utilized, and cancer types investigated.

## 4. Gut Microbiota’s Influence on Patients’ Response and Sensitivity to CPIs

### 4.1. Effect of Host Microbiota Profile on the Response to CPIs in Humans

As previously discussed, the gut microbiota profile plays an important role in influencing the response of animal models to CPIs. This relationship has also been investigated in humans, in an attempt to find a correlation between specific microbial profiles and improved responses to CPIs. There currently exists a significant amount of evidence from human cohorts suggesting that the gut microbiota constitute a key player in modulating the response to CPIs in humans [75]. The first prospective study assessing the role of gut microbiota in determining the response to CPIs in metastatic melanoma patients concluded that CPIs’ responders were more likely to have gut microbiota profiles enriched with *Bacteroides caccae*, irrespective of the type of CPI used [28]. On the other hand, in a study performed by Chaput et al. in 2017, metastatic melanoma patients with baseline gut microbiota enriched with *Faecalibacterium* and other *Firmicutes* had higher progression-free survival (PFS) and overall survival (OS) when treated with ipilimumab, compared to those with gut microbiota profiles rich in *Bacteroides* (*p* = 0.0039 and 0.051 respectively) [76]. Similar studies in metastatic melanoma patients associated specific microbial profiles with enhanced responses to CPIs [27,64]. The influence of microbiota was not only restricted to melanoma patients. In the context of renal cell carcinoma, the abundance of *Akkermansia muciniphila* in stool at the time of diagnosis was associated with better response to CPIs [77]. Similar findings were also reported by Routy et al., who assessed stool samples from patients with renal cell and non-small cell lung carcinoma [30]. Moreover, the abundance of Lactobacilli and Clostridia was shown to prolong time to treatment failure in non-small cell lung cancer patients treated with CPIs [78]. On the other side, some bacterial profiles were associated with resistance to CPIs. For example, gut microbiota profiles rich in *Ruminococcus obeum* and *Roseburia intestinalis* were shown to be associated with poor response to CPIs in metastatic melanoma patients [64]. In addition, while *Alistipes putredinis*, *Bifidobacterium longum* and *Prevotella copri* were enriched in the guts of responders to PD-1 blockade in a Chinese cohort of non-small cell lung cancer patients, *Ruminococcus* species were associated with decreased response to anti-PD-1 therapy [79]. Overall, there is not a great deal of overlap between specific bacterial taxa and their relative influence on the response to CPIs in different studies, and no taxon to date has been consistently associated with clinical response to CPIs [28,75,76]. In fact, the different outcomes between the different studies in humans may be attributed to several different factors. These include differences in the techniques used to analyze samples and in the reference databases used for analysis between the different studies [75]. Geographic, lifestyle, and dietary differences may also play a role in this regard [75]. All this being said, it is clearly apparent that microbial profiles do have an influence on the response of cancer patients treated with CPIs. While the big majority of the studies confirmed that microbial diversity is mostly associated with a better response to CPIs, it is important to develop standardized approaches to better to account for the differences encountered in the various studies [25,75].

### 4.2. Factors Associated with Impairment of Patients’ Microbial Profile during Cancer Treatment

Given that gut microbiota was proven to be involved in shaping the response to cancer treatment, attention should be paid to the potential daily interventions that might influence this microbial profile during the treatment of those patients. First of all, antibiotics constitute one of the major classes of drugs that are frequently prescribed to this population of patients [80]. This class of drugs in turn plays a major role in modulating the composition of the gut microbiota, consequently influencing the response of cancer patients to CPIs [81]. In a systematic review by Pierrard et Seront, the use of antibiotics was shown to be associated with a decreased efficacy of CPIs [82]. In fact, regardless of the cancer type, patients treated with CPIs and receiving antibiotics had a lower objective response rate, progression-free and overall survival, compared to their counterparts who did not receive antimicrobial agents [82]. The impact of antibiotics on the response to CPIs could be explained by several mechanisms [82]. Most importantly, by selectively modulating the bacterial flora in the gut, antibiotics could favor the selection of specific microbial species that might confer resistance to treatment with CPIs [13,73]. As such, while it might be difficult to select a specific class of antibiotic as a culprit in this setting, it has been proven that broad-spectrum antibiotics are mostly involved in conferring this resistance to CPIs [82]. In fact, Ahmed et al. have shown that broad-spectrum antibiotics were associated with a decrease in the objective response rate to CPIs, a relationship that was not proven with the use of narrow-spectrum antibiotics [83]. This deleterious effect of antibiotics on CPIs may last for a period of several months, highlighting the importance of weighing risks and benefits when prescribing antibiotics to cancer patients treated with CPIs [30,82,84].

In addition to antibiotics, other concomitant medications routinely used during the treatment of cancer patients also have an impact on the gut microbiota. Proton pump inhibitors (PPIs) are associated with a decreased diversity and taxonomical changes in the gut microbiota [85]. In fact, PPIs could modulate the gut microbiota by changing the gastric pH and selecting specific bacterial species [86,87]. Data regarding the effect of PPIs on the response to CPIs remains controversial. In a multicenter observational study involving 1012 treated with CPIS, PPIs were shown to be associated with a significantly higher risk of disease progression and death [88]. Similar results were also reported in several studies raising concerns about the use of PPIs in cancer patients treated with CPIs [87,89]. On the other side, other studies failed to conclude a causal relationship between the use of PPIs and worse outcomes in patients with cancer treated with CPIs [90,91]. Given this controversy, it remains debatable whether the use of PPIs could influence the response to CPIs, and carefully assessing the need of PPIs should be done before randomly or routinely prescribing them to cancer patients.

**Figure 4:**
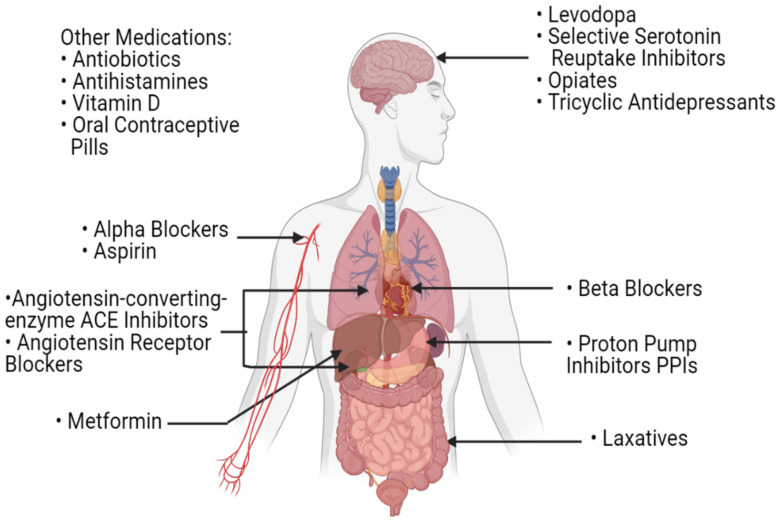
A list of commonly used medications in the daily routine of patients with cancer. These medications can influence the gut microbiota and potentially affect the host’s response to immune checkpoint inhibitors.

Laxatives, which are routinely used by cancer patients, also play a role in the modulation of the gut microbial profile [81]. In fact, stool transit time, stool consistency and bacterial load per stool sample all contribute to the shaping of the bacterial profile of the gut [92,93]. As such, by affecting those parameters, the frequent use of laxatives in patients with cancer could potentially have an influence on their gut microbiota and consequently, on their response to CPIs’ treatment. While this correlation has not been extensively studied, Katayama et al. have shown that patients with non-small cell lung cancer with constipation and frequent laxative need had lower overall survival and time to treatment failure when treated with CPIs as compared to those with regular bowel movements [94]. Other drugs that are routinely used by all patients of old age, including serotonin-reuptake inhibitors, angiotensin-converting enzyme inhibitors, metformin, beta-blockers and many others, were shown to have an effect on the composition of the gut microbiota (Figure 4) [81]. As such, concomitant drugs used during the treatment of cancer patients can have an influence on their response to treatment with CPIs by modulating their gut microbiota. In this setting, and as more investigations are needed, polypharmacy should be carefully dealt with in patients with cancer, especially those treated with CPIs.

## 5. Gut Microbiota to Overcome Resistance to CPIs in Humans

### 5.1. Mechanisms through Which the Gut Microbial Profile Controls the Response to CPIs

Now that we have mounting evidence concerning the role that gut microbiota plays in the extent of the response to CPIs, it is important to assess at the cellular level the mechanisms that are used by those microbes allowing them to exert such a role. To start with, gut microbiota contributes to the local and systemic education of the immune response [95].

First of all, dendritic cells DCs constitute a major link between the microbial profile of the gut and their ability to shape the immune response and consequently, the response to CPIs [95]. Recent research from animal models and humans concluded that the activation of macrophages and DCs is under the control of the gut microbiota [96]. In fact, pathogen-associated molecular patterns (PAMPs) from the gut microbiota are recognized by Toll-like receptors (TLRs) of the DCs leading to their activation [95]. In their turn, activated DCs contribute to the activation of the innate immune response [95]. Moreover, activated DCs can migrate to the mesenteric lymph node and prime the adaptive immune response against tumor cells [95]. This is made possible through the activation of CD-8+ T cells and Th1 cells with the consequent upregulation of INF-γ, TNF-α and Granzyme B in patients treated with anti-PD1/PD-L1 and anti-CTLA-4 [95]. In a study by Vetizou et al., orally feeding melanoma mice models with *B. fragilis* accelerated the maturation of DCs in the tumor microenvironment and enhanced the Th1 response in the tumor-draining response, allowing them to overcome the resistance to anti-CTLA-4 [73].

In addition to activating DCs, bacterial profiles play a role in controlling the levels of regulatory T cells (Tregs) in the blood of patients, consequently affecting their anti-tumor immune response [96]. Tregs actively suppress immune response by suppressing lymphocytes [97]. Patients with good responses to CPIs have microbial profiles that favors low levels of Tregs, unlike those with poor responses, who have microbial profiles favoring high levels of Tregs in the peripheral blood [96]. As such, Smith et al. postulated that favorable bacterial profiles decrease peripheral Tregs, allowing for a stronger response to anti-PD-1 blockade [98].

Furthermore, and as mentioned above, beneficial bacteria play an essential role in increasing cytokine production [96]. While the loss of INF-γ signaling was shown to induce resistance to anti-CTLA-4 treatment, the introduction of beneficial bacteria was shown to be associated with significantly higher levels of this cytokine in the tumor-draining lymph nodes of melanoma mice models [13,99]. Routy et al. have also shown that the restoration of anti-PD1 response by fecal microbiota transplant from responders was possible through an increase in IL-12 and the recruitment of CD4+ T lymphocytes in the tumor microenvironment [30]. Increases in IL-12 levels potentially enhance the response to CPIs through an increase in INF-γ production allowing a heightened natural killer (NK) and T cells response and favoring a TH1 phenotype with a strong antibody-dependent cytotoxicity [100,101]. In addition, the secretion of chemokines may be also influenced by gut microbiota composition. In a study by Cremonsi et al., the exposure of colorectal cancer cells to gut flora increased the production of CCL-5, CCL20 and CXCL10 by 70-, 19- and 12-fold, respectively, and the increase in chemokines was microbial-load dependent [102]. This increase in chemokines is attributed to a microbiota-dependent increase in the migration of tumor-infiltrating lymphocytes to the tumor microenvironment [102].

This communication between the microbiota and the immune system is possibly mediated through a system of microbial metabolites [95]. Microbes within the gut ferment dietary fibers, leading to the production of SCFAs. These are absorbed through the intestinal epithelium and transmitted to the T cells via G-protein coupled receptors, enhancing their anti-tumor activity [103,104]. Butyrate, a SCFA, for example was shown to induce the differentiation of CD8+ T cells [105]. In addition, SCFAs were shown to play a role in the differentiation and proliferation of Tregs, promoting immunotherapy response [96]. Moreover, the level of SCFA in the stools of patients with solid tumors were significantly higher in those who responded to nivolumab compared to those who did not [105]. As such, it has been concluded that SCFA-producing microbiota enhance the response to CPIs [95].

### 5.2. Clinical Use and Modulation of Microbiota to Overcome Resistance to CPIs

Now that the role of gut microbiota in the function of the patient’s immune system and his response to CPIs is confirmed, it became essential to build on this recently acquired knowledge in order to clinically overcome the resistance to CPIs faced by some patients with cancer [106]. As such, the modulation of the gut microbiota could potentially constitute an effective method in this regard [106].

#### 5.2.1. Fecal Microbiota Transplant (FMT)

By definition, FMT allows the direct transfer of a solution of fecal matter from a donor into the intestinal tract of a recipient in order to directly change his gut microbiota composition, trying to elucidate a specific health benefit [107]. In this way, an entire microbial ecosystem is transferred to the recipient, allowing a robust engraftment of the introduced bacteria as a whole community rather than a single species that might be prone to competition by the recipient’s initial microbiota [108]. The concept of FMT was initially established in 1958 by Eisman et al., who concluded in a case series that the transplant of functional microbiota from healthy individuals could help in treating *Clostridium difficile* pseudomembranous colitis by re-establishing the healthy microbiota in infected patients [109]. Later on, FMT was introduced to the world of hematologic malignancies as a potential treatment of post-hematopoietic stem cell transplant pseudomembranous colitis in patients with lymphoma [110,111,112]. Preclinical studies on mice models showed promising results concerning the use of FMT to improve responses to CPIs [27,30,64]. In fact, melanoma mice models that received FMT from previous CPI responders had higher CD8+ infiltration within the TME and better responses to PD-1 blockade, compared to those who received their FMT from non-responders [27,64]. Baruch et al. were among the first to clinically assess the safety and efficacy of FMT in patients with anti-PD-1 refractory metastatic melanoma [113]. In their phase I clinical trial, 3 out of 10 included patients responded to anti-PD-1 post FMT, including one complete response [113]. Similar findings were also reported in a phase I clinical trial by Davar et al., where 6 out of 15 patients with anti-PD-1 refractory melanoma treated with FMT from responders had clinical benefit [114]. Most importantly, both studies showed that treatment with FMT was able to induce favorable changes in the tumor microenvironment, including increases in CD8+ immune cell infiltrates, decreased frequency of IL-8-expressing immunosuppressive myeloid cells and favorable gene-expression profiles [113,114]. Responders were also shown to have distinct proteomic and metabolomic signatures that were regulated by the gut microbiota [114]. Several clinical trials assessing the role of FMT in the response of patients to CPIs are currently ongoing. While its safety has been demonstrated even in immunocompromised patients, attention should be paid in the future regarding the possible complications of FMT, as some cases of FMT were reported to induce bacteremia through an unclear mechanism [112,115]. Three important points should be considered prior to FMT administration: the screening of the bacterial constituents of the FMT, the removal of harmful pathogens (bacteria, viruses, or parasites), and the possible isolation of cultivation of less abundant but beneficial microorganisms [116].

#### 5.2.2. Probiotics

Unlike FMT, probiotics have been used for years [117]. They constitute a collection of active microorganisms involved in improving the health status of their recipient by restoring a healthy intestinal flora [117]. Certain combinations of probiotics were shown to enhance the immune response of their recipients, as noted by an increase in immune-favorable blood biomarkers (CD3, CD4 and CD8 T cells) in patients receiving probiotics after chemoradiation therapy [118]. The role of probiotics in enhancing response to CPIs has brought promising results in preclinical studies involving animal models. In a study by Sivan et al. involving melanoma mouse models, the administration of oral *Bifidobacterium,* in conjunction with anti-PD1 immunotherapy, nearly abolished tumor cells’ growth [13]. This combination was shown to increase the activity of dendritic cells and the recruitment of CD8+ T cells with the tumor microenvironment [13]. Similar results were also reported by Routy et al., where the oral administration of *Akkermansia muciniphila* restored the efficacy of anti-PD1 therapy though an Il-12 dependent mechanism allowing the recruitment of CD4+ T cells to the tumor microenvironment [30]. In addition to their interaction with the immune system, probiotics were found to secrete several metabolites that have anti-tumor properties, including inorganic polyphosphates, competence and sporulation factors, ferrichromes and other peptides, including P40 and P75 [119]. This remarkable success of probiotics in improving the response to CPIs is currently under investigation in human clinical trials. In fact, probiotic colonization in humans is more challenging, compared to in mice, given the huge diversity of their microbial ecosystems [120]. While using antibiotics before the administration of probiotics could help in improving colonization, one study has shown that antibiotics use hindered the establishment of a diverse microbial ecosystem upon the administration of probiotics [121]. Given the variation in composition between different industries and the absence of results from human clinical trials to date, the use of probiotics off-trial during the treatment of patients with cancer should not be encouraged [122,123]. In addition, despite being easy to use, affordable and easily accessible, probiotic use still faces many challenges including the variable rate of engraftment in the setting of competing commensals and the potential to lower the beneficial gut diversity [108]. Recent technological advances allowed the development of designer probiotics (mimicking FMT but with a consistent composition) and commensal bacteria probiotics (previously unculturable), both of which showed promising results [123,124].

#### 5.2.3. Prebiotics

Prebiotics are inactive food supplements selectively favoring the growth of specific beneficial microbial species in the gut of their recipient [125,126]. They are mainly made up of dietary fibers, whose metabolism leads to the formation of short chain fatty acids that favor an acidic intestinal environment [127]. Such an environment selectively allows the growth of beneficial bacteria, including *Lactobacilli* and *Bifidobacteria* [127]. In addition, they enhance the production of resistant starch by the gut microbiota, with the subsequent formation of butyric acid, a metabolite with anti-cancer and anti-inflammatory properties [128]. As such, being able to affect the gut microbiota composition, several clinical trials are assessing their role in influencing responses to CPIs [108]. However, concerns have been raised, as such interventions might affect the overall diversity of the gut microbiota, a factor that is usually considerable favorable in the response to CPIs [108].

#### 5.2.4. Diet and Lifestyle

Diet is an additional factor regulating the gut microbial profile [129]. It has been shown that patients who consume fiber-rich diets have different gut microbial profiles than those who consume fat-rich diets [129]. In addition, studies have shown that specific dietary interventions had some minor effects on microbiota and anti-tumor immune responses [130]. By shaping the composition of the gut microbiota, diet could potentially influence the response of patients with cancer to CPIs [131]. However, diet-induced microbiota changes were directly reversed after the discontinuation of the specific diet plans, indicating that diet interventions should be consistent in order to potentially detect a favorable long-term anti-tumor response [108].

Exercise also plays a role in modulating gut microbiota and subsequent response to CPIs [132]. As compared to control groups, rugby players were found to have more diverse gut microbial profiles, with lower levels of inflammatory biomarkers [133]. In addition, by decreasing the levels of lactic acid, exercise may induce a better response to CPIs by increasing immune-cell infiltration within the tumor microenvironment and regulating the expression of PD-L1 in tumor cells [134,135].

Finally, sleep quality was shown to affect the composition of the gut microbiota [136]. In fact, late bedtime was shown to disrupt gut microbial diversity [131]. In addition, studies in mice confirmed that recurrent sleep disruptions modulated the gut microbiota [137]. As such, by modulating the gut microbiota, sleep quality is an additional lifestyle factor that can influence the anti-tumor response to CPIs.

## 6. CPIs, Angiogenesis, and Microbiota: A Future Perspective

There is now a growing body of evidence to suggest that the gut microbial profile is an essential modulator of the response to CPIs. In addition, gut microbiota were shown to be involved in the process of angiogenesis and the development of vasculature [138]. For example, bacterial lipopolysaccharides were shown to activate vascular endothelial growth factor (VEGF) and consequently, promote angiogenesis [139]. Moreover, tumor resident bacteria were shown to disrupt the gut vascular barrier, allowing the dislocation of gut microbiota into systemic circulation [140]. This dislocation promoted the “maturation” of a premetastatic niche at distant organs, favoring the metastatic cascade in colorectal cancer [140]. On the other hand, angiogenesis is proven to be involved in modulating the response to CPIs. In this regard, the combination of anti-VEGF and PD-L-1inhibitors was shown to increase progression-free and overall survival in patients with unresectable hepatocellular carcinoma and metastatic renal cell carcinoma [141,142]. As such, a complex interaction exists between gut microbiota, angiogenesis, and response to CPIs. In this regard, combining CPIs with antiangiogenic therapy and microbiota modulation could offer potential therapeutic benefits in the future, enhancing the response to CPIs.

## 7. Conclusions

In conclusion, there exists strong preclinical and clinical evidence that the gut microbiota plays a fundamental role in shaping the response of tumors to treatment with CPIs. This influence is made possible through various direct and indirect mechanisms that allow these microorganisms to influence the immune system’s composition and function and consequently, shape the immune response. As such, the modulation of this microbial profile through multiple interventions including FMT, pro and prebiotics, as well as lifestyle changes, could potentially constitute an essential step towards overcoming resistance to CPIs in many cancer patients with poor responses to immunotherapy.

## Figures and Tables

**Table 1:** Preclinical studies assessing the gut microbiota and the response to immune checkpoint inhibitors.

Bacteria	Immunotherapy	Target Tumor	Main Findings	Reference
*Bifidobacterium* spp.	PD-L1 mAb	Melanoma	*Bifidobacterium*-spp.-treated mice had significantly improved tumor control compared to non-*Bifidobacterium*-treated mice.	[13]
*Ruminococcaceae* Bacteroidales	PD-1 mAb	Melanoma	Higher levels of the *Ruminococcaceae* family in the feces of ICI responders compared to a high abundance of the Bacteroidales order in non-responders.	[25]
*Bacteroidales* spp.	PD-1 mAb ± CTLA-4 mAb	Colon adenocarcinoma	Impaired response to PD-1 mAb ± CTLA-4 mAb in gnotobiotic- or antibiotics-treated mice Recovered immune response to immunotherapy after oral gavage of human bacteria strains mix (11-mix).	[63]
*Akkermansia muciniphila*	PD-1 mAb ± CTLA-4 mAb	MCA205, LLC and RET tumor-bearing mice	Increased levels of *Akkermansia muciniphila* in ICI responders Reversal of resistance to anti-PD-1 blockade therapy in antibiotic-pretreated mice after FMT from ICI responders.	[30]
*Bifidobacterium longum* *Collinsella aerofaciens* *Enterococcus faecium*	PD-L1 mAb	Melanoma	Enhanced T-cell responses and improved efficacy of anti-PD-L1 therapy with oral supplementation of responders’ fecal material to germ-free mice.	[64]
*B. bifidum*	PD-1 mAb	Colon and Lewis Lung Carcinoma	Specific *B. bifidum* strains conveyed synergistic association with anti-PD-1 therapy to decrease the tumor burden. Reduced anti-PD-1 therapy efficacy seen in antibiotics treated mice. Reconstitution with favorable *B. bifidum* strain and PD-1 antibodies reversed resistance in PBS-treated or antibiotics-treated mice. INFγ receptor blockade abrogated the tumoricidal effects of anti-PD-1 and anti-PD-1 plus favorable *B. bifidum* strain. Upregulation of genes associated with lymphocyte activation, peptidoglycan synthesis and INFγ production.	[65]
*Akkermansia muciniphila* *Bacteroides*	PD-1 mAb	MSS-type colorectal cancer	Poor anti-tumor response in colistin-treated mice compared to a medium response in vancomycin-treated mice. High levels of *Akkermansia muciniphila* conveyed enhanced anti-tumor response in vancomycin-treated mice, compared to *Bacteroides* enrichment in non-responding mice. Favorable microbiota promoted the expression of metabolic and synthetic pathways, such as glycerolipid metabolism.	[68]
*Bacteroides cellulosilyticus* *Bacteroides caccae*	PD-1 mAb	Glioma	Strong positive response to anti-PD-1 therapy with decreased tumor growth and prolonged survival in responder mice that showed abundance in *Bacteroides cellulosilyticus* Bacteroides caccae Rescue experiments failed to reverse resistance to anti-PD-1 immunotherapy after responder fecal transplant in antibiotics-treated non-responder mice.	[69]
*Bacteroides thetaiotaomicron* *Bacteroides fragilis*	CTLA-4 mAb	Melanoma	Impaired anti-tumor immunity in germ-free and antibiotics-treated mice. Recovery of anti-tumor response of anti-CTLA-4 via oral feeding with *Bacteroides* spp. and *Burkholderia* spp. Enhanced response to CTLA-4 blockade with FMT from patients with increased *Bacteroides* spp. levels.	[73]
